# The Genetic Basis of Inbreeding Avoidance in House Mice

**DOI:** 10.1016/j.cub.2007.10.041

**Published:** 2007-12-04

**Authors:** Amy L. Sherborne, Michael D. Thom, Steve Paterson, Francine Jury, William E.R. Ollier, Paula Stockley, Robert J. Beynon, Jane L. Hurst

**Affiliations:** 1 Population and Evolutionary Biology Group, School of Biological Sciences, University of Liverpool, Liverpool L69 7ZB, United Kingdom; 2 Mammalian Behaviour and Evolution Group, Department of Veterinary Preclinical Science, University of Liverpool, Leahurst, Neston CH64 7TE, United Kingdom; 3 Centre for Integrated Genomic Medical Research, University of Manchester, Manchester M13 9PT, United Kingdom; 4 Proteomics and Functional Genomics Group, Department of Veterinary Preclinical Science, University of Liverpool, Crown Street and Brownlow Hill, Liverpool L69 7ZJ, United Kingdom

**Keywords:** EVO_ECOL

## Abstract

Animals might be able to use highly polymorphic genetic markers to recognize very close relatives and avoid inbreeding [1, 2]. The major histocompatibility complex (MHC) is thought to provide such a marker [1, 3–6] because it influences individual scent in a broad range of vertebrates [6–10]. However, direct evidence is very limited [1, 6, 10, 11]. In house mice (*Mus musculus domesticus*), the major urinary protein (MUP) gene cluster provides another highly polymorphic scent signal of genetic identity [8, 12–15] that could underlie kin recognition. We demonstrate that wild mice breeding freely in seminatural enclosures show no avoidance of mates with the same MHC genotype when genome-wide similarity is controlled. Instead, inbreeding avoidance is fully explained by a strong deficit in successful matings between mice sharing both MUP haplotypes. Single haplotype sharing is not a good guide to the identification of full sibs, and there was no evidence of behavioral imprinting on maternal MHC or MUP haplotypes. This study, the first to examine wild animals with normal variation in MHC, MUP, and genetic background, demonstrates that mice use self-referent matching of a species-specific [16, 17] polymorphic signal to avoid inbreeding. Recognition of close kin as unsuitable mates might be more variable across species than a generic vertebrate-wide ability to avoid inbreeding based on MHC.

## Results

Our experimental design met a set of stringent requirements to establish whether mice use major histocompatibility complex (MHC) and/or major urinary protein (MUP) to avoid inbreeding. First, genetic recognition and mate preferences need to be demonstrated against normally variable genetic and environmental backgrounds typical of natural populations. We therefore used wild house mice (*Mus musculus domesticus*) rather than genetically homogeneous laboratory mice that are hybrids of three *Mus* subspecies derived from an extremely small pool of founders [18, 19] and have been subject to strong artificial selection for ease of breeding in the laboratory [4, 20]. Second, previous studies examining kin recognition and inbreeding avoidance in mice have used only MHC types derived from laboratory strains (e.g., [3–5, 21, 22]). The role of MUPs in this context has not been examined previously, probably because of the lack of variation in MUP patterns within and between laboratory strains ([23], J.L.H. and R.J.B., unpublished data, and S.A. Cheetham, personal communication). This is in dramatic contrast to wild house mice, in which individual variation in the pattern of urinary MUPs is the main basis for individual recognition through scent [12, 13, 15, 24]. To properly reflect natural variation at these highly polymorphic gene complexes, we used founder mice that carried a large range of MHC and MUP haplotypes derived directly from wild animals. Third, in natural populations, animals that share the same genotype at a highly polymorphic marker such as MHC or MUP will be closely related and share many other alleles across the genome. In order to test whether inbreeding avoidance is driven by the sharing of MHC and/or MUP haplotypes (inherited independently) on naturally variable genetic backgrounds, we controlled for background relatedness in our analysis and design and thus the sharing of other alleles across the genome. Finally, to ensure natural behavior and mate choice, we allowed mice to breed freely in very large seminatural enclosures without intervention or disturbance.

In house mouse populations, dominant males defend small territories in which they monopolize mating opportunities [25], and extraterritorial matings by females with neighbor territory owners are frequent [4, 26]. Animals that remain in the same local population are thus likely to encounter unfamiliar half sibs that share the same father but different mothers in addition to full sibs that share both father and mother. To model this, we released four replicate populations of half-sib and full-sib outbred house mice (each derived from one father and several unrelated mothers with full sibs reared only by their mothers; see the Supplemental Experimental Procedures in the Supplemental Data available online) into four very large seminatural enclosures (each 250 m^2^ with abundant cover and food). Tissue samples for genotyping were obtained from all F1 founders (33 female, 48 male) prior to release and from their parents, allowing us to establish the separate MHC and MUP haplotypes of each heterozygous founder. The combinations of males and females available as potential mating partners covered the full range of possible MHC and MUP haplotype sharing (Table S1). After 15 weeks, when founding females could have reared up to three litters to independence, animals were captured from each population and F2 offspring of the original founders (n = 483) were genotyped with 40 microsatellite markers so that parentage and MHC and MUP haplotypes could be independently established. From these data, we determined for each F1 founder female the minimum number of successful matings with each available male (based on recovered independent offspring) and the number of offspring captured from each female-male pair.

Consistent with inbreeding avoidance, we found a deficit in the overall frequency of successful matings between full sibs (Table S2), although the effect was too weak to differ significantly from random mating per female (Table 1: multinomial logistic model 1, p = 0.08). We found no evidence for disassortative mating on the basis of MHC haplotype sharing (Table 1: model 2), i.e., there was no evidence for fewer successful matings between mice that shared either one or both MHC haplotypes relative to those that shared no haplotypes (Table S2; Figure S1). By contrast, MUP sharing had a strong and highly significant effect on the likelihood of successful mating (Table 1: model 3, p = 0.005; Figure S1). Specifically, there was no deficit when only one MUP haplotype was shared, but there were many fewer matings between mice that shared both MUP haplotypes (complete match) than expected under random mating conditions (Table 1: model 4, p < 0.002). The same trend was evident across all four populations (Table S1). We confirmed that this effect was due to MUP sharing between males and females rather than inherent differences in male quality because there was no difference in the overall mating success of males represented in full MUP sharing dyads (mean ± standard error of the mean [SEM] matings per male with any female: 4.4 ± 0.6, n = 23) compared to the success of other males (3.8 ± 0.5 matings per male, n = 25; t_46_ = −0.70, p = 0.49). The strong deficit in successful matings between mice of the same MUP genotype also accounts for the weak deficit in matings between full sibs (Table 1: model 5 versus model 4, p = 0.6) because full sibs were much more likely than half sibs to share both MUP haplotypes (Table S1).

It has been hypothesized that familial (behavioral) imprinting on maternal haplotypes would allow animals to avoid inbreeding with a greater proportion of kin than the use of self-haplotype sharing alone [21]. Behavioral imprinting on maternal haplotypes (MHC, MUP, or other genetic loci) should result in a general avoidance of full sibs compared to paternal half sibs because all full sibs carry a maternal haplotype at each potential genetic marker. However, the inclusion of overall relatedness (full versus paternal half sib) as well as MUP genotype sharing in the model provides no better explanation of the likelihood of mating than does MUP genotype alone (Table 1: model 5 versus model 4, p = 0.6). Specific analysis of female behavioral imprinting on maternal MHC haplotypes (Table 1: model 6) or maternal MUP haplotypes (Table 1: model 7) also failed to significantly improve explanation of the likelihood of mating above the direct sharing of MUP genotype alone (see the Supplemental Experimental Procedures for further discussion).

There were fewer offspring per successful mating with full sibs than random expectation (Table 2: model 1, p = 0.02), consistent with the lower viability of offspring due to inbreeding depression [2, 27] or with postcopulatory sperm or embryo selection by females [28]. However, there was no evidence that MHC or MUP sharing between parents per se reduced the viability of offspring from successful matings. Thus, it is unlikely that the strong deficit in successful matings between mice of the same MUP genotype (above) was due to lower viability of these offspring. The small deficit in offspring from matings between animals sharing both MHC haplotypes or both MUP haplotypes (Table S2) was nonsignificant (Table 2) and was due to the fact that most dyads in these two categories were full sibs. Full sibs had significantly fewer offspring per mating regardless of MHC or MUP sharing. Similarly, the MHC and MUP genotypes of all F2 offspring followed the expected pattern of Mendelian inheritance (Table S2), indicating that MHC and MUP homozygotes and heterozygotes had equal viability with no evidence for differential postcopulatory selection.

Why should mice avoid mates that share both MUP haplotypes but not those that share one haplotype? In our experiment, the sharing of both MUP haplotypes was a good predictor of whether a potential mate was a full sib or not: 31% of full-sib dyads shared both MUP haplotypes versus only 5% of half-sib dyads (Table S1). However, because 45% of full sibs and 54% of half sibs shared one MUP haplotype, this was a poor guide to whether a potential mate was a full sib. A simulation of populations containing different numbers of haplotypes shows this to be a general rule and not just a consequence of the mix of founders used in the current experiment (Figure S2). Even when there are many haplotypes in the population, the sharing of one or zero haplotypes provides very little information on whether a potential mate is likely to be a full sib or a nonrelative, whereas the sharing of both haplotypes considerably increases the likelihood that animals are full sibs. Mice thus avoid mating when shared MUP type reliably indicates very close relatedness.

## Discussion

The use of MUP alone was sufficient to explain inbreeding avoidance in this study. Although the deficit in mating between mice of the same MUP type was very strong, there was no evidence that animals used genetic markers other than MUP to improve their level of inbreeding avoidance. Further, there was no evidence to support the previously untested hypothesis that animals could increase the range of relatives avoided by imprinting on the separate haplotypes carried by their mother [21]. Such a strategy would also result in the rejection of any unrelated animals that happen to share one maternal haplotype. Notably, animals should only avoid related partners when there is a high inbreeding load [29, 30], which is only likely to occur between very close relatives within outbred populations. As we have shown, the sharing of both haplotypes (but not one) is a reliable indicator of very close relatedness. Nonetheless, in fully outbred populations, most full sibs will not share both MUP haplotypes (only 31% shared both MUP haplotypes in the current study), a situation that applies to any highly polymorphic loci, including MHC. Although this does not exclude a large proportion of close relatives, avoidance between mice sharing both MUP haplotypes might be sufficient to drive widespread avoidance between very close relatives under natural conditions. Theoretical modeling predicts that female discrimination against related males in polygynous mating systems will drive male-biased dispersal from natal areas even when discrimination is relatively weak [29]. Consistent with this, the high level of dispersal among young males in house mouse populations [31] will have a general effect of separating close relatives of the opposite sex, reducing inbreeding even between pairs that do not share MUP type.

The use of MUP as a genetic marker for inbreeding avoidance (whether a pre- or postcopulatory mechanism) will promote genome-wide heterozygosity that includes MHC. Despite widely held assumptions in the literature that MHC-based scents are used by females to avoid inbreeding and promote MHC heterozygosity [1, 5–9, 22, 32], direct evidence is surprisingly limited. Correlations between MHC dissimilarity and mate choice within natural populations [11] might arise through the use of other non-MHC cues because of the normal correlation between MHC and genome-wide similarity. Some congenic laboratory mice that differ only at MHC (though see [7]) show disassortative mating preferences, but others do not [3, 5]. Moreover, the relevance of such studies to the behavior of normal mice is highly questionable [4, 20]. Genetic recognition also needs to be demonstrated against the variable genetic and environmental backgrounds typical of natural populations [15, 33]. Studies of hybrid laboratory mice crossed with wild mice to derive subjects with laboratory-derived MHC haplotypes but with 50% of their genomes from wild mice [4, 21, 34] reveal a deficit of MHC homozygous offspring. However, the design and interpretation of these studies remains controversial [1, 5, 35, 36]. Offspring were typed only for MHC, so parentage could not be assigned and, crucially, parental differences in overall relatedness and other genes that might contribute to inbreeding avoidance could not be assessed. Because the inbred strains used to derive MHC and half of the genome also had two different MUP types, the deficit in MHC homozygous offspring could have arisen from a correlation between MHC and MUP types in the derivation of the founder lines. Genetic variation in these hybrids was also likely to be substantially reduced compared to wild mice. Further research is needed for us to understand whether familial imprinting on MHC, MUP, or other genes is important in much more inbred populations when potential mates might share a complete match to maternal genotypes or perhaps in extreme situations when only MUP identical mates are available. However, such situations could be very unusual because MUP variation persists even in an isolated population with low overall genetic variation that regularly undergoes genetic bottlenecks [37]. Our findings further highlight the limitations of using laboratory strains in mate-selection studies when mice are unable to use their normally variable MUP signals for either kin or individual recognition. Not only will these key signals be unavailable in tests of preference, but the lack of exposure to natural variation in individual scents during the rearing of inbred animals might strongly impact the development of normal recognition processes.

Two broad outcomes derive from this study. First, it challenges the widely held assumption that MHC scents provide a general mechanism across vertebrates to avoid inbreeding and promote MHC heterozygosity. This idea has arisen largely from studies of laboratory mice under extremely abnormal genetic, social, and environmental conditions. Instead, normal wild mice use a set of species-specific urinary proteins to avoid inbreeding that has evolved to provide optimized characteristics for effective signaling through their urine scent-marking system. Given the importance of reliable identity information for mate selection and reproductive success, we should expect animals to evolve signals that are most appropriate for reliable communication in that species. The only known function of MUPs is in scent communication, and they are produced at very high abundance in urine by mice of both sexes but with particularly strong investment by adult males [14]. These proteins provide direct information about genetic identity at several different levels in mice (species, sex, individual, and kinship) ([12, 15, 16, 38], present study) in addition to integrating identity and status information through bound low-molecular-weight volatile pheromones [13, 14, 39]. Individual MUP signatures can easily be identified regardless of other genetic variation between wild mice [12, 15], confirmed by the disruption of recognition when a recombinant MUP is added to wild mouse urine [12]. The fact that mice failed to use MHC scents in addition to MUP when this could have improved inbreeding avoidance suggests that MHC scents might not be easily recognized under naturalistic conditions, despite the ability of mice to discriminate MHC scents in laboratory tests in which background variation is suppressed [7, 8, 40]. Although MUPs are produced in large quantity and are very resistant to degradation, providing a persistent signal that can be left in the environment as well as for direct communication [13], the short peptide ligands bound by MHC [8] lack the structural features for proteolytic resistance and are therefore highly susceptible to both endoproteolytic and exoproteolytic attack. This would limit their reliability and persistence in scent signals. Although this does not mean that MHC has no role in communication through scent, this might be more limited than previously assumed. MUP-like orthologs are likely to be present in other species that use scent communication because proteins can provide direct information on genetic identity, but as yet there has been little investigation of alternative polymorphic markers beyond MHC. Initial studies indicate that MUP-like lipocalins are expressed by many rodents with a high degree of species specificity and diversity of expression [17]. However, species that rely on visual, acoustic, or other forms of communication might encode genetic identity information through alternative polymorphic mechanisms.

The second general implication is that the ability to recognize kin as a mechanism to avoid inbreeding might be more variable across species than previously considered. Mate-selection mechanisms to avoid the deleterious consequences of inbreeding are only likely to be important where kin encounter each other as adults [2]. Because house mice often live at high density in family-based social groups to exploit locally abundant resources [25], the need to recognize kin to avoid inbreeding will be relatively strong. However, the extent of polymorphism in MUP-like urinary proteins varies considerably between *Mus* species [17]. Notably, MUPs are not polymorphic in *Mus macedonicus* [16], a closely related grassland species that lives at much lower densities where individuals are much more widely dispersed than house mice, and thus the need to recognize kin to avoid inbreeding is likely to be considerably less. The ability to avoid inbreeding through kin recognition between adults might be restricted to those species that have evolved specific polymorphic communication signals to achieve this rather than a general ability across vertebrates. Some species might also rely on familiarity of signals learnt during rearing rather than self-referent phenotype matching if relatives reliably interact in the absence of nonkin [41]. The understanding of these issues promises to provide considerable insight into the importance of kin recognition for the avoidance of inbreeding and maintenance of genetic heterozygosity in different social systems. It is timely to extend the scope of research into such polymorphic signaling systems beyond the MHC.

## Figures and Tables

**Table 1:** Multinomial Logistic Models for Frequency of Mating

	β	Log Likelihood	Likelihood Ratio (LR) Statistic^a^	df	P^b^
Model 1: Full-Sib Avoidance					
full sib	−0.36	−491.07	3.42	1	0.08 (one tailed)

Model 2: MHC Sharing					

one MHC haplotype	0.36	−490.63	4.31	2	0.27
both MHC haplotypes	0.12				

Model 3: MUP Sharing					

one MUP haplotype	0.15	−483.60	18.36	2	0.005
both MUP haplotypes	−1.36				

Model 4: Full MUP Sharing					

both MUP haplotypes	−1.44	−484.04	17.48	1	0.002

Model 5: Relatedness and Full MUP Sharing					

full sib	−0.12	−483.86	17.84	2	0.65^c^
both MUP haplotypes	−1.38				

Model 6: Maternal MHC Imprinting and Full MUP Sharing					

one MHC haplotype match between male and female's mother	−0.33	−482.61	20.34	2	0.18^c^
both MUP haplotypes	−1.31				

Model 7: Maternal MUP Imprinting and Full MUP Sharing					

one MUP haplotype match between male and female's mother	−0.11	−483.76	18.04	2	0.54^c^
both MUP haplotypes	−1.35				

Summary of observed and expected frequencies for each category shown in Table S2.

a Compared to null model (log likelihood = −492.78).

b Probabilities calculated by random permutation of data (n = 10,000).

c Comparison to model 4.

**Table 2:** Multinomial Logistic Models for Number of Offspring per Mating

	β	Log Likelihood	LR Statistic^a^	df	P^b^
Model 1: Full-Sib Avoidance					
full sib	−0.44	−497.90	7.69	1	0.02^c^ (one tailed)

Model 2: MHC Sharing					

one MHC haplotype	−0.12	−499.24	5.02	2	0.25
both MHC haplotypes	−0.49				

Model 3: MUP Sharing					

one MUP haplotype	0.11	−500.59	2.33	2	0.52
both MUP haplotypes	−0.34				

Summary of observed and expected frequencies for each category shown in Table S2.

a Compared to null model (log likelihood = −501.75).

b Probabilities calculated by random permutation of data (n = 10,000).

c Model 1 would also be significant at p < 0.05 in a two-tailed test.

## Supplemental Data

Document S1. Experimental Procedures, Four Figures, and Four Tables
