# Supplementary material for: The Genetic Basis of Inbreeding Avoidance in House Mice
Source: Curr Biol. 2007 Dec 4;17(23):2061–6. doi: 10.1016/j.cub.2007.10.041 (PMC2148465; doi:10.1016/j.cub.2007.10.041)
Supplement: Document S1. Experimental Procedures, Four Figures, and Four Tables [file mmc1.pdf]

Supplemental Data
The Genetic Basis of Inbreeding Avoidance in House Mice

Amy L. Sherborne, Michael D. Thom, Steve Paterson, Francine Jury, William E.R. Ollier, Paula Stockley, Robert J. Beynon, and Jane L. Hurst

Supplemental Experimental Procedures

Subjects
The breeding plan simulated a naturalistic situation in which a dominant male sires offspring from several females, resulting in a local population of full-sib and paternal half-sib mice. Thus, animals had a choice of mating with full or half siblings and with individuals with whom they shared zero, one, or two MHC or MUP haplotypes (see Figure S3 for a schematic illustrating the sharing of haplotypes between full and half sibs). This design was used because inbreeding depression is only a high risk between very close (first order) relatives in outbred populations, and inbreeding depression must exceed a substantial threshold before any avoidance behavior is expected to evolve [S1]. Because outbred full sibs are twice as closely related as half sibs, their offspring have twice the risk of inheriting homozygous deleterious recessive alleles, as well as having lower heterozygosity. Factors other than inbreeding depression influence the selection of genetically compatible mates among less closely related mates [S2], and unrelated or more distant relatives are unlikely to share MHC or MUP types. For each of four separate populations, we bred founders by housing a single male house mouse (*Mus musculus domesticus*) with two sets of three unrelated females (each for 14 days) in breeding cages (40 × 23.5 × 12.5 cm). Mice were captive-bred F1–F3 unrelated animals from an outbred captive colony derived from wild ancestors captured from five different populations in the northwest of England, UK. Females were housed singly prior to parturition (cages 40 × 23.5 × 12.5 cm) so that full-sib mice were familiar during rearing but half sibs met only after release into enclosures as adults. For each population, three to five females produced F1 offspring sired by the same male (see Table S1). Offspring were separated into single sex and sibship groups at weaning (4 weeks) and released into enclosures when they were 48–65 days old. Prior to release, all founders were given a subcutaneous radiofrequency identification (RFID) tag for individual identification, and a small tissue sample was taken from the tip of the tail (1–2 mm) under general anesthesia (halothane) for genotyping. Genetic samples were also taken from the founders' parents to allow the individual MHC and MUP haplotypes carried by each founder to be identified. There was no attempt to eliminate endemic parasites or viruses from our stock colony, which was regularly supplemented with wild-caught animals, with the exception of a screening program for the elimination of lymphocytic chorionic meningitis virus. The animal procedures used in this study were approved by the UK Home Office and the Animal Welfare Committee of the University of Liverpool, UK.

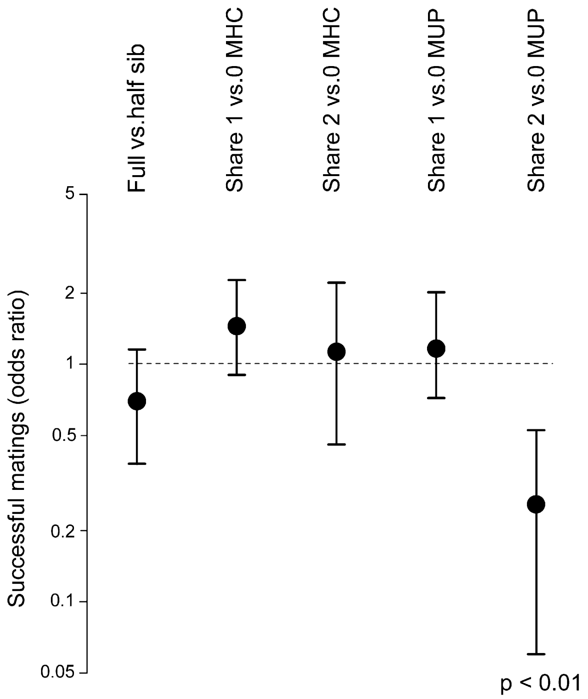

Figure S1. Odds Ratio of Successful Matings between Different Dyads
Successful matings per female with a full sib versus a half sib and when sharing one or two MHC or MUP haplotypes versus sharing no haplotypes, estimated from a multinomial logistic model. An odds ratio of 1 indicates no mating bias (pre- or postcopulatory), and an odds ratio of less than 1 indicates avoidance. Ninety-five percent confidence intervals generated by bootstrapping.

Population Enclosures
Founders sharing the same sire were released simultaneously into one of four very large (25 × 10 m) outdoor enclosures, each containing substantial long grass ground cover. The very large size of the enclosures ensured that all of the founders had the opportunity to establish territories. Vegetative cover was supplemented with 30 nest boxes spread throughout each enclosure, although mice generally preferred to nest in the grass; ten concrete block shelters (45 × 45 × 35 cm) were added after 12 weeks when rain became heavy. Ten food and water stations, spaced evenly around the outer walls of each enclosure, provided ad libitum food (Lab Diet 5002 Certified Rodent Diet) and water. Sheet-aluzinc walls prevented escape or contact between populations (1.3 m high with concrete foundations for the prevention of climbing or burrowing), and wire mesh upper walls and roof prevented predation. Mice were allowed to breed undisturbed before being live trapped 15–19 weeks after founder release. Each founder female could have reared up to three F2 litters to independence over this period that could be clearly distinguished as F2 offspring (see below). Upon capture, founder mice were identified from their RFID tags and housed in captivity for use in further studies. All founders were recaptured and so were available as possible mates throughout the experiment. Sex, weight, and age class (adult, subadult, juvenile) were recorded for all animals, and a urine sample was obtained for further studies on MUP phenotyping. Nonfounders were then culled humanely under halothane anesthetic, and tail snips were taken for genotyping. Blood and gut samples were also taken for other studies.

MHC and MUP Genotyping
We established the MHC and MUP haplotypes of the founders for each population by using eight microsatellite markers across the MHC region on chromosome 17 and eight microsatellite markers surrounding the MUP region on chromosome 4 (Table S3). Full details of DNA extraction, polymerase chain reaction (PCR) amplification, and product analysis are given in reference [S3]. The patterns of alleles present in the founders were compared to their parents so that linked alleles in the same haplotype and any crossover events could be identified. All F1 founders were heterozygous for MHC and MUP except for four females in population B, which were MUP homozygous.

To genotype the F2 offspring of the F1 founders, we selected three to four of the MHC markers and three to four MUP markers that

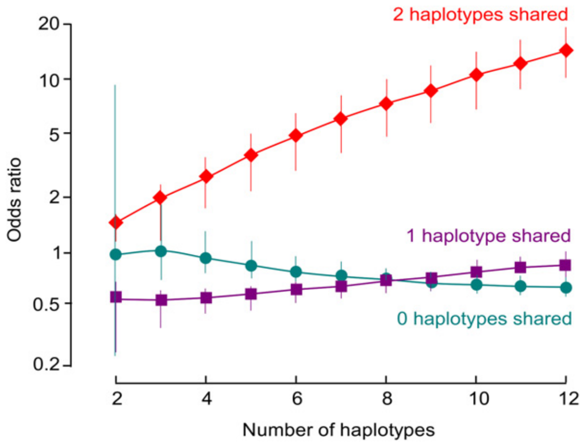

Figure S2. Simulation Model

The odds in favor of a male and female being full sibs given the number of haplotypes that they share at a polymorphic complex. This is equivalent to the Bayes factor, i.e., the extent to which genotype information modifies a prior odds ratio to give a posterior odds ratio. Circles indicate no haplotypes shared, squares indicate one haplotype shared, and diamonds indicate two haplotypes shared. Ninety-five percent confidence intervals are shown. An odds ratio of 1 indicates that the posterior odds ratio is unaffected by the genotype information. The odds in favor of a male and female being full sibs when two haplotypes are shared is analogous to the concept of genotype matching described by Grafen [S13], and here we extend this to the case for sharing only one haplotype.

reliably discriminated each haplotype within a population (different sets of markers were used for each population, see Table S3). To check that MHC homozygotes were correctly identified and were not due to poor primer amplification (leading to only one of two heterozygote alleles being detected by mistake), we ran an additional marker for any animals initially classified as MHC homozygotes. This confirmed homozygosity in all cases.

Parentage Assignment

Because parentage could only be assigned reliably to the F2 offspring of the founders, we excluded all animals that might have been F3 s on the basis of their weight, sex, and date of capture. If founders mated immediately after introduction to the enclosures, F2 offspring from the first litters could have started breeding 9 weeks after founder release (wild mice in enclosures rarely breed before 6 weeks old, plus a 3 week gestation period) and produced offspring up to 3 weeks old when trapping began. We therefore excluded all mice that were 3 weeks old or less at week 15, according to sex-specific growth curves for F2 mice subsequently bred in captivity from the same half-sib founders (n = 19 litters weighed at weekly intervals from age 3 weeks). This left 497 offspring across the four enclosures. DNA was extracted from tail snips with 96-well AGOWA mag DNA Isolation kits and a Hamilton Microlab STAR robot. We selected 24 microsatellite markers from the Mouse Genome Informatics site (MGI 3.51) spread across the genome and not linked (>50 cM) to either each other or the MUP or MHC regions (Table S4). The forward primer for each marker was 5′-fluorescently labeled with 6-FAM, NED, PET, or VIC among one of three size groups such that 12 markers could be pooled into a single run. PCR amplification was conducted in 10 μl reactions containing 10 ng of DNA, 0.5 μM of each primer, and 5.0 μl of 2X BioMix Red reaction mix (Bioline [London, UK]) and incubated at 95°C for 2 min, and 30 cycles of 95°C for 30 s, 52°C–58°C (depending on the primer) for 2 min, then 72°C for 30 s followed, with a final extension at 72°C for 10 min. The PCR reactions were then diluted 50- to100-fold and multiplexed in formamide with GeneScan LIZ500 size standard (Applied Biosystems), and size was determined with an ABI PRISM 3100 DNA analyzer and GeneMapper v3.0 software (Applied Biosystems). Parentage analysis was carried out with CERVUS v3.0 [S4] so that individuals

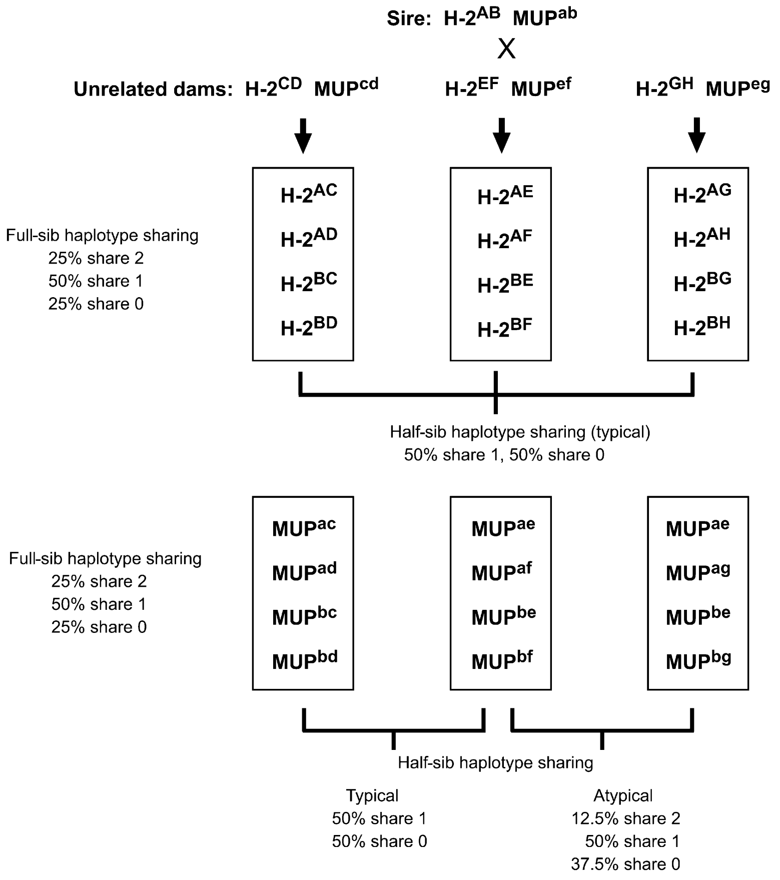

Figure S3. Expected Haplotype Sharing among Founders for MHC or MUP

Full-sib mice derived from unrelated heterozygous parents with different haplotypes for MHC (termed H-2 in mice) and MUP on average will share two haplotypes (25% of full-sib dyads), one haplotype (50%), or no haplotypes (25%). Among half sibs, 50% inherit the same haplotype from their father, whereas 50% share no haplotype. Occasionally, unrelated parents share a haplotype through common descent from the same populations (“MUP^{e}” in the example shown), resulting in a small proportion of half sibs that share two haplotypes. Haplotypes, denoted in uppercase letters for H-2 and lowercase letters for MUP, were derived from wild mice and do not refer to known haplotypes from laboratory strains.

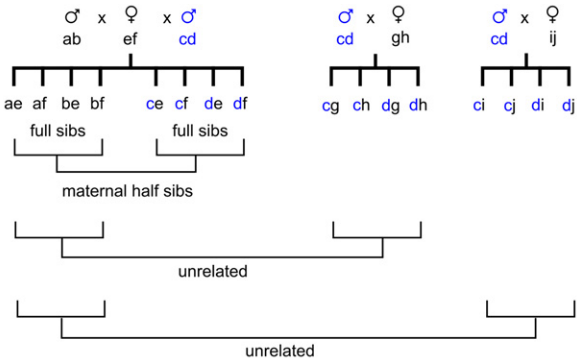

Figure S4. Behavioral Imprinting on Parental Haplotypes

Schematic illustrating haplotype sharing when a female (“ef”) mates with more than one male (“ab,” “cd”), such as two unrelated territory owners (letters represent MHC or MUP haplotypes, or alleles of other highly polymorphic genes, and show possible combinations among offspring). Familial imprinting on all haplotypes carried by littermates would lead to the incorrect recognition of many unrelated animals as relatives. In the example, offspring of female ef would recognize any animals carrying haplotypes a–f as relatives (depending on haplotypes represented in the litter). This would result in offspring of male ab × female ef incorrectly recognizing male cd and all his offspring as relatives. In house mice, where related females nest communally and each mate with local male territory owners, familial imprinting on littermates could mean that most of the local dominant males together with their offspring are avoided as mates, even when they are not relatives. Familial imprinting only on maternal haplotypes would allow the recognition of all full sibs and maternal half sibs as relatives [S7].

could be assigned to a parent pair with maximum-likelihood methods. All assignments were carried out blind to MHC and MUP type. We then checked for any incompatibilities between parentage and MHC and MUP genotypes. We found mismatches in only 2.8% of cases (14 out of 497 offspring typed). All of these were below the median weight for the youngest mice classified as F2 and, because these were probably F3 offspring, they were excluded from the data set.

Mating Assignment

Because individual offspring are not independent data points, we determined the minimum number of successful matings that must have occurred to explain the offspring captured. For each founder female, we plotted offspring weight against capture date and used the sex-specific growth curves from captive F2 together with paternity to assign offspring to separate litters (females could have had a maximum of three litters that were over 3 weeks old by the start of trapping). We took the most conservative approach and only assigned offspring from the same father to separate matings if they were very unlikely to have come from the same litter (based on their weights and a maximum litter size of nine for wild house mice). This resulted in the assignment of 193 successful matings across the four enclosures, with all females producing at least two litters. Notably, 67% of litters were sired by more than one male. Because matings were assigned blind with respect to MHC or MUP genotypes, any errors in assignment could not cause any bias in our analysis.

Statistical Analysis

We used the statistics package R v2.3.1 (www.r-project.org) to fit logistic multinomial models to the data with likelihood methods [S5]. Two response variables were analyzed: (1) the number of successful matings made by a female from each of a set of available males and (2) the number of offspring produced from each of a set of observed matings. The log likelihood can be given by

$$\left.\begin{aligned} \ln L &= \sum_i \sum_j y_{ij} \ln P_{ij} \\ P_{ij} &= \frac{\exp\left(\left(\mathbf{x}_{ij} - \mathbf{x}_{i1}\right)\beta\right)}{\sum_j \exp\left(\left(\mathbf{x}_{ij} - \mathbf{x}_{i1}\right)\beta\right)} \end{aligned}\right\}, \qquad \text{(S1)}$$

in which y_{ij} is the observed number of matings or offspring from sire *j* and dam *i*, and x_{ij} is a vector of explanatory variables that describes sire *j* with respect to dam *i* (i.e., whether the sire is a half or full sib of the dam and whether the dyad shares one, two, or no MHC or MUP haplotypes, respectively). β is the vector of coefficients, fitted by the maximization of the log likelihood with numerical optimization. In the case of two-tailed tests, the significance of explanatory variables was determined by the comparison of LR statistics (i.e., twice the log likelihood ratio for a pair of nested models) to a distribution generated by a permutation procedure in which the genotype or relatedness of the dam was randomly reordered with respect to the set of available males. This permutation approach is more conservative than the comparison of LR statistics against a chi-square distribution because it controls for potentially inflated Type I errors that might arise from the repeated preference of a dam to a particular sire, unconnected to the relatedness or genotype of the sire with respect to the dam. In the case of one-tailed tests, the significance was determined by the comparison of the coefficient fitted to the observed data with the corresponding distribution generated by the permutation procedure.

Familial Imprinting

Animals might avoid inbreeding not only by the comparison of their own genotype to that of potential mates but also by behavioral imprinting on the scents of close relatives during rearing. Previous evidence for MHC effects on sexual preferences suggest that mice prefer mates of different MHC type from the parental odors experienced in the nest rather than those differing from self [S5–S8], and it has been hypothesized that negative imprinting on MHC-determined odors would allow females to avoid inbreeding with a greater proportion of kin than self inspection alone [S7]. Because extra-pair matings and multiple paternity occur frequently in house mice [S9, S10], and related females often rear their offspring communally [S11], indiscriminate imprinting on all nest mate haplotypes would be very error prone and result in avoidance of many unrelated mates (Figure S4). However, imprinting only on maternal haplotypes would allow the recognition and avoidance of all full sibs and maternal half sibs [S7]. To test this, we compared the likelihood of mating according to whether or not males had an MHC or MUP haplotype that matched one of those carried by a female’s mother. Note that all males necessarily shared one MHC and one MUP haplotype with the mother of full sib sisters, whereas some also shared one MHC or MUP haplotype with mothers of paternal half sibs through common ancestry from the same populations. Because these were outbred populations, no males shared both MHC haplotypes with a female’s mother, and only a very small number of dyadic combinations shared both MUP haplotypes (2.8%, all full sibs). We also checked for any evidence of negative imprinting on maternal haplotypes among males [S8] but found no effects.

Simulation Modeling

We investigated, across a range of possible populations, whether a male sharing 0, 1, or 2 haplotypes with a female was a good guide as to whether he was a brother or not. We modeled the odds in favor of a male and female being full sibs compared to being unrelated given the number of haplotypes that they share at a polymorphic locus,

$$\frac{\Pr(\text{sibs}|x)}{\Pr(\text{unrelated}|x)} = \frac{r}{1-r} \cdot \frac{p(x|\text{sibs})}{p(x|\text{unrelated})}, \qquad \text{(S2)}$$

i.e., the posterior odds ratio of a male and female being full sibs or not is the product of the prior odds ratio (in the simplest case derived from the proportion, *r*, of full sibs that a female encounters) and the Bayes factor [S12], which is the odds ratio of the probabilities that a male and a female will share *x* haplotypes given that they are or are not sibs. The Bayes factor, therefore, is the quantity of interest because it is a measure of whether the extra genotypic information gained from odor cues have increased or decreased the relative odds of a potential mate being a brother.

In order to estimate the odds ratio $p(x|\text{sibs})/p(x|\text{unrelated})$, we performed separate simulations for different numbers of alleles at a locus, ranging from two alleles up to 12 (Figure S2). For each simulation, we drew 1000 allele frequency distributions at random. From each allele frequency distribution, we estimated $p(x|\text{sibs})$

Table S1. Summary of F1 Founders and F2 Offspring in Each Population

	Population				
	A	B	C	D	Total
F1 Founders	6 female, 7 male (3 litters)	12 female, 18 male (5 litters)	7 female, 12 male (3 litters)	8 female, 11 male (3 litters)	33 female, 48 male (14 litters)
MHC haplotypes	6	9*	5	6*	16
MUP haplotypes	7*	9	7	7*	20
Number of Female:Male Dyads					
Full sib	18	36	14	28	96
Half sib	24	180	70	60	334
MHC Haplotype Sharing between Founders (% Full-Sib Dyads/% Half-Sib Dyads)					
both	27.8/0	38.9/0.6	57.1/4.3	25.0/6.7	35.4/2.4
one	50.0/62.5	41.7/59.4	42.9/95.7	53.6/45.0	46.9/64.7
none	22.2/37.5	19.4/40.0	0/0	21.4/48.3	17.7/32.9
MUP Haplotype Sharing between Founders (% Full-Sib Dyads/% Half-Sib Dyads)					
both	27.8/0	22.2/3.3	35.7/2.9	42.9/13.3	31.3/4.8
one	44.4/54.2	52.8/59.4	50.0/48.6	32.1/46.7	44.8/54.5
none	27.8/45.8	25.0/37.2	14.3/48.6	25.0/40.0	24.0/40.7
F2 offspring genotyped	99	192	101	91	483
Number of Matings Observed (Expected) According to MHC Haplotypes Shared					
both	2 (3.4)	9 (5.7)	1 (5.3)	5 (4.6)	17 (19.1)
one	20 (16.6)	50 (45.1)	41 (36.7)	19 (19.0)	130 (117.3)
none	8 (10.0)	22 (30.2)	0 (0)	16 (16.4)	46 (56.5)
Number of Matings Observed (Expected) According to MUP Haplotypes Shared					
both	0 (2.9)	3 (5.9)	0 (3.2)	3 (8.8)	6 (20.7)
one	22 (15.4)	51 (48.1)	19 (20.7)	22 (16.4)	114 (100.5)
none	8 (11.7)	27 (27.1)	23 (18.2)	15 (14.8)	73 (71.8)

"*" Indicates that one founder inherited a crossover haplotype, combining alleles from two haplotypes. For analysis, this was regarded as a match for either of the original haplotypes.

and $p(x|\text{unrelated})$ from 250 samples in which we picked two genotypes randomly according to Hardy-Weinberg expectations, set these as parents, and then generated a daughter from these parents and determined (1) the proportion of her brothers sharing 0, 1, or 2 alleles and (2) the proportion of unrelated males in the population sharing 0, 1, or 2 alleles with her, which we approximated from Hardy-Weinberg expectations.

Supplemental References

S1. Kokko, H., and Ots, I. (2006). When not to avoid inbreeding. Evolution Int. J. Org. Evolution *60*, 467–475.

S2. Bateson, P. (1983). Optimal outbreeding. In Mate Choice, P. Bateson, ed. (Cambridge: Cambridge University Press), pp. 257–278.

S3. Cheetham, S.A., Thom, M.D., Jury, F., Ollier, W.E.R., Beynon, R.J., and Hurst, J.L. (2007). The genetic basis of individual-recognition signals in the mouse. Curr. Biol. *17*, 1771–1777.

S4. Kalinowski, S.T., Taper, M.L., and Marshall, T.C. (2007). Revising how the computer program CERVUS accommodates genotyping error increases success in paternity assignment. Mol. Ecol. *16*, 1099–1106.

S5. Jobson, J.D. (1992). Categorical and Multivariate Methods, *Volume II*, Applied Multivariate Data Analysis. (New York: Springer).

S6. Penn, D.J. (2002). The scent of genetic compatibility: Sexual selection and the major histocompatibility complex. Ethology *108*, 1–21.

S7. Penn, D., and Potts, W. (1998). MHC-disassortative mating preferences reversed by cross-fostering. Proc. R. Soc. Lond. Ser. B-Biol. Sci. *265*, 1299–1306.

S8. Yamazaki, K., Beauchamp, G.K., Kupniewski, D., Bard, J., Thomas, L., and Boyse, E.A. (1988). Familial imprinting determines H-2 selective mating preferences. Science *240*, 1331–1332.

S9. Potts, W.K., Manning, C.J., and Wakeland, E.K. (1991). Mating patterns in seminatural populations of mice influenced By MHC genotype. Nature *352*, 619–621.

S10. Dean, M.D., Ardlie, K.G., and Nachman, M.W. (2006). The frequency of multiple paternity suggests that sperm competition is common in house mice (*Mus domesticus*). Mol. Ecol. *15*, 4141–4151.

S11. Manning, C.J., Wakeland, E.K., and Potts, W.K. (1992). Communal nesting patterns in mice implicate MHC genes in kin recognition. Nature *360*, 581–583.

S12. Bernardo, J.M., and Smith, A.F.M. (2000). Bayesian Theory (Chichester, United Kingdom: Wiley).

S13. Grafen, A. (1990). Do animals really recognize kin? Anim. Behav. *39*, 42–54.

Table S2. Summaries of Observed and Expected Frequencies among F2 Offspring

**Number of Matings**			
Relatedness	Half Sib	Full Sib	
Observed	159	34	
Expected[a]	149.16	43.84	
MHC Sharing	None	One Haplotype	Both Haplotypes
Observed	46	130	17
Expected[a]	56.53	117.35	19.12
MUP Sharing	None	One Haplotype	Both Haplotypes
Observed	73	114	6
Expected[a]	71.76	100.51	20.73
**Number of Offspring**			
Relatedness	Half Sib	Full Sib	
Observed	414	69	
Expected[b]	395.92	87.08	
MHC Sharing	None	One Haplotype	Both Haplotypes
Observed	135	311	37
Expected[b]	124.78	310.57	47.65
MUP Sharing	None	One Haplotype	Both Haplotypes
Observed	159	314	10
Expected[b]	164.48	304.67	13.85
**Offspring Genotype**			
MHC	Homozygous	Heterozygous	
Observed	94	389	
Expected[c]	96.25	386.75	
MUP			
Observed	77	406	
Expected[c]	83.50	399.50	

[a] Expected on the basis of the proportion of dyads of each type available for each female.
[b] Expected on the basis of matings of each type per female.
[c] Expected on the basis of parental genotypes and number of offspring per mating.

Table S3. Microsatellite Markers Used for MHC and MUP Genotyping

MHC Marker	Location	Forward Primer	Reverse Primer	Size	Repeat	Label	Comments	F2*
D17Mit230	17 b2 32.3 Mb	5′-TGACATAAACCTC TGGCTTCC-3′	5′-CCAGCCCATCTAAA GTCATTTC-3′	288	CA	ned	duplicated in all mice	A, B, C, D
D17Mit22	17 B2 33.9 Mb	5′-GCATTAGATAGAGA GTAGATGGGTTG-3′	5′-ATGGATGGCGAGAA TGAGAC-3′	216	GT	hex	used	A, B, D
D17Mit231	17 B2 34.1 Mb	5′-GCCTCAGCAAGAC CCTAAAC-3′	5′-ACTCCTCCTTTTCCC TCTCC-3′	285	GT	hex	used	
D17MIT13	17 B2 34.6 Mb	5′-TGCAGGCAAGATC CAAGAAG-3′	5′-GAAAGAGGGTGTCG ATGCTC-3′	239	GT	hex	used	
D17Nds3	17 B3 34.8 Mb	5′-CAGCCTTAATGGG TCTGGTC-3′	5′-ACAGAGGGGAAGAG GAAAGC-3′	222	CT	ned	used	B
D17Mit47	17 B3 35.8 Mb	5′-CTGAGACCAGTGCA GTGGAA-3′	5′-TTTTTCAATATGTGAG CATGTGC-3′	238	CA	fam	used	C
D17Mit24	17 B3 37.0 Mb	5′-ACCTCTCACCTCTC TCTGTG-3′	5′-GCAAGTTTAGGGATCT TTTCTCC-3′	131	CA	fam	used	A, B, C, D
**MUP Marker**	**Location**	**Forward Primer**	**Reverse Primer**	**Size**	**Repeat**	**Dye**	**Comments**	**F2***
D4NDS6	4 B3 52.8 Mb	5′-CGGGGAAGGTTGT TTGTTTG-3′	5′-AGGCCAGCAATGTAG AAAGG-3′	240	GT	ned	used	
D4Mit139	4 B3 55.2 Mb	5′-TCAAACTGGGAAG AGCCAAG-3′	5′-GCCGTAGAAGAGAAG TAATTTTTCC-3′	149	GT	hex	used	D
D4Mit241	4 C1 55.7 Mb	5′-TTTCCAGTGTTGT CCAGAGC-3′	5′-AAGGCAAATCACTAG GTGCTG-3′	219	CA	hex	used	D
D4Mit288	4 C1 56.8 Mb	5′-ACATTCAGCAAA GACTGAGCAC-3′	5′-TGCCATTTGTTATAGA CCATGC-3′	168	CA	ned	used	A, B
D4 mit164	4 C1 59.5 Mb	5′-AACACATATATACC AAGGCAGCAC-3′	5′-ATTTCCACCCTGTCCA CTCC-3′	142	CA	fam	used	A, B, C
D4Mit243	4 C1 59.6 Mb	5′-AGCCCTACTGATT GCTCTCC-3′	5′-TGGAAAGTTGAAAAC CACTGC-3′	168	CA	fam	used	A, B, C, D
D4Mit217	4 C1 59.7 Mb	5′-ACTCAATTAGGTT GTTCAGATAGCC-3′	5′-GGCACTTGCTGCCA CATC-3′	246	GT	hex	used	
D4Mit17	4 C1 62.8 Mb	5′-GCCAACCTCTGTG CTTCC-3′	5′-CCTCTGACATCCAC ACACATC-3′	138	GT	fam	used	A, B, C, D

"*" Indicates that after MHC and MUP haplotypes in F1 founders and their parents were identified with all listed markers, only a subset were necessary for the identification of haplotypes in F2 offspring for each of populations A–D.

Table S4. Microsatellite Markers Used for Parentage Assignment

Marker	Position (cM)	Location (Mb)	Forward Primer	Reverse Primer	Size	Label
D1Mit58	1 8.3	9.7	5′-GGACTGGCAATCCTCTTGTC-3′	5′-GCACGTTAGAGAGTGGGCTC-3′	254	VIC
D1Mit155	1 112.0	196.1	5′-ATGCATGCATGCACACGT-3′	5′-ACCGTGAAATGTTCACCCAT-3′	252	NED
D2Mit405	2 68.9	149.7	5′-TGATTATATCTTGGAATACACGTGTG-3′	5′-CTGTGTAGCAAAACAGTTTATGGC-3′	84	6-FAM
D3Mit1	3 11.2	0.3	5′-TGTGCACAGGGGTACATACA-3′	5′-TCATTTTCTTCCTCCCCCTC-3′	143	VIC
D3Mit163	3 87.6	157.3	5′-TGGATACATACATATACATGGAAATGC-3′	5′-TTTCTCCAGACCCATGAACC-3′	143	6-FAM
D4Mit171	4 6.3	22.6	5′-CAGGTGTAATAATGGTTTTTTGACC-3′	5′-CATATTAAATAAACACAGCAGCACG-3′	318	PET
D4Mit310	4 71.0	147.5	5′-TCTCCACGTGTGTGCCTTAG-3′	5′-TGAAAGCACTCTGCAGACTCA-3′	117	PET
D5Mit25	5 61.0	111.8	5′-AACACACCTCCATACTGGTCG-3′	5′-GGCTAACTGAAATTGTTTTGTGC-3′	234	NED
D6Mit105	6 45.5	108.6	5′-CTGTCTCCACTACTTCTATTCCTGG-3′	5′-CAAAAGCCTTATATATTACACCTCACC-3′	237	6-FAM
D7Mit253	7 55.0	115.3	5′-TGTGGGTGCAACCAAATG-3′	5′-TTTGGTGATATAGATACTAGGTGTGTG-3′	89	NED
D8Mit29	8 33.0	70.7	5′-CCCTAGTGTATACATAGAGGGGTG-3′	5′-TCTTTGTGTTGTGATGTGTTGTAA-3′	109	VIC
D9Mit12	9 55.0	99.4	5′-ATTCAAGGGGCAGTACACAT-3′	5′-TGGTCCTGGTAAAACTGCCT-3′	96	6-FAM
D10Mit80	10 4.0	11.5	5′-CAAAAAAAACCCTGATTCTACCA-3′	5′-GTGTGCATATGGCAGTAACTTTG-3′	154	NED
D10Mit98	10 59.0	102.0	5′-TCAGGCATCTAGTGAGATGATCC-3′	5′-CCCATAGATGCAGGGGTG-3′	150	VIC
D11Mit63	11 2.0	17.1	5′-GCCCACAACTTTGTGTCCTT-3′	5′-TTGACCATGCTCCTCATCAG-3′	139	PET
D11Mit300	11 68.0	111.3	5′-TTTGGCTGTGATAAAACAAAACA-3′	5′-TTGAGTTTTGATTTTGTATGTGGG-3′	145	6-FAM
D12Mit4	12 34.0	79.9	5′-ACATCCCCAGCTCTTGTTTG-3′	5′-AAACCAAACCAAAGAAGCTTAGG-3′	201	PET
D13Mit77	13 73.0	118.0	5′-TCTTTGAAGTCCCTTTCAAAGC-3′	5′-ATAGCACTGCACTCATGCTCA-3′	275	VIC
D14Mit212	14 13.5	36.2	5′-AACATGTGCACTGGAACAATG-3′	5′-TCATTTATCAATTTACTTTGGTGAGG-3′	102	PET
D15Mit161	15 69.2	97.9	5′-TCTGTTTTGTTTGTTCGTTTGC-3′	5′-TAAAATCTCCCTGTATACAAGTCTGTG-3′	99	NED
D16Mit71	16 70.5	98.0	5′-TAGAAAATCTTCAAATAGGATCTGTTC-3′	5′-GAGCATTTCCCTTTTACCTGG-3′	154	PET
D17Mit94	17 45.9	75.8	5′-TGGAGAGGCATCCAACTCTC-3′	5′-TTCCTCTTAGTCCACCTTTTGC-3′	146	NED
D18Mit33	18 44.0	70.0	5′-GCATGTCGTATCCATAAACATACG-3′	5′-ATGCGGGCTTGACTTCTG-3′	140	VIC
D19Mit70	19 51.0	50.8	5′-AAAATATCAGGGCATGGTGC-3′	5′-GGGTTATTAGGAAAATTTATGTTGTG-3′	195	6-FAM
